# Modelling climate variabilities and global rice production: A panel regression and time series analysis

**DOI:** 10.1016/j.heliyon.2023.e15480

**Published:** 2023-04-14

**Authors:** Masha Joseph, Stephan Moonsammy, Harold Davis, Devin Warner, Ashley Adams, Temitope D. Timothy Oyedotun

**Affiliations:** a Department of Environmental Studies Faculty of Earth and Environmental Sciences, University of Guyana, P. O. Box 10 1110, Turkeyen Campus, Greater Georgetown, Guyana; b Department of Geography Faculty of Earth and Environmental Sciences, University of Guyana, P. O. Box 10 1110, Turkeyen Campus, Greater Georgetown, Guyana; c Hydrometeorological Service, Ministry of Agriculture, Georgetown, Guyana

**Keywords:** Climate variability, Precipitation, Rice production, Temperature, Trend analysis

## Abstract

Climate change threatens agriculture and it remains a present global challenge to food security and Sustainable Development Goals. The potential impact on the supply of crops such as rice is seen as a major food security issue that requires intervention on several fronts. The literature on rice production, climate variations and climate change show several studies outlining various impacts on rice supply as a result of variations in temperature and rainfall. This study intends to further explore the impacts on rice production caused by temperature changes and rainfall variation by analyzing and modelling the production of rice by the top rice-producing countries globally. A time series of the national rice production and yield along with national average annual temperature and rainfall were sourced for 15 major rice-producing countries. The trends of the time series were then compared between the rice productivity variables and temperature and rainfall. A panel regression model was also developed to further assess the relationship between rice production and temperature and rainfall. The time series showed that rice production and yield are increasing for the majority of the countries analyzed. The panel regression model however showed that continued increase in temperature can result in decreased production of rice and that rainfall volume directly impacts rice output and therefore shows rice production is highly susceptible to flooding and drought events caused by climate variabilities.

## 1 Introduction

The IPCC [1] defines climate variability as the variation of climate on all spatial and temporal scales beyond that of individual weather events. With the ongoing multi-sectoral challenges of climate change, the vulnerabilities across regions have become more evident, particularly in the agricultural sector [2,[3], [4]^5^,^6^,^7^,^8^]. Observations in the variations in temperature and precipitation are commonly used as indicators for climatic changes and climatic events [9]. These climatic changes often lead to an increase in extreme weather-related events such as flooding or drought which significantly impact global agricultural production [10,11,5,12,13]. The impacts of abnormal temperature and rainfall variations in the production of crops, and in the intensification of threats to global food security are well documented in the literature [e.g. Refs. [[4], [14],^15^,^16^,^17^,^18^,^19^,^20^]].

One of the main concerns about the climatic impacts on food production is the impact on cereal grains which are one of the major sources of food and nutrients globally [21]. Grains are not only used by over 50% of the global population, also an estimated 40% is used as livestock feed [22,23,21]. Given its extensive usage for human and livestock consumption, grains have a primary role in achieving both local and global food security [24]. For instance, the literature postulates that a decline in the production of rice as a major grain source can compromise global food security [25,26,27]. The national impacts of climate variations on food security have been recognized by some countries such as China, Malaysia and India [28,29,30,31,32,33,34].

Rice is the single largest grain consumed globally, with the demand for it expected to increase by 25% by 2030 [35,36,21]. Approximately 90% of production occurs in the Asian continent, with 75% coming from China, Indonesia and India alone [37,24]. It has medicinal, cosmetic and other agricultural uses, as well as potential for development into functional food products [38,39,40]. Different varieties of rice may have distinct health and cosmetic benefits for an individual [36] emphasizing the versatility and wide range of maretable products derived from rice production.

Rice is a semi-aquatic crop requiring saturated fields at the seedling stage and dry conditions at harvesting [41,42]. Factors influencing rice production include the area harvested, the methods of cultivation and the use of modern technological advancements [2,[43], [44]]. The conditions needed for growing rice are also often linked to localized weather patterns [45,46]. Several studies have postulated the negative effects on rice production as a result of variations in temperature and rainfall [47,48,49]. For example, Mahdu [50] who conducted a study in Guyana and Ayinde et al. [51] who did their study in Niger, both reported declines in rice production due to increased temperature and rainfall despite these studies occuring in two different continents.

To minimize losses due to inclement weather conditions and improve production, farmers have adopted improved drainage and irrigation practices and have begun to integrate the use of climate-smart agriculture (CSA) [52,53,54,43,17,55]. Studies have shown that temperature and rainfall changes can have a positive impact on rice production in some countries [56]. According to Huang et al. [57] and Zhang et al. [58] with the use of climate smart agricultural practices, rice production will continue to increase in China. Additionally, Mahmood et al. [59] concluded that with an estimated increase of 1.5 °C to 3 °C in temperature, the rice yields in the Punjab Province of Pakistan are expected to increase in the next three decades. It was also noted by Waltthal et al. [60] that with temperature increases, Northern rice-producing regions may also see an increase in production as opposed to the rice cultivated in the Tropical and Semi-Tropical regions. In light of the conclusions in the literature, it is necessary, that the rice-producing countries across the continents be aware of the relationships between climate variations and rice production, [50].

### 1.1 Temperature effects on rice production

Singh and Awais [31] highlighted that rice is one of the more sensitive crops to climate variations. These variations are accompanied by other unpredictable physical and biotic conditions, such as increased pests and diseases and diminishing soil quality [61,62,63]. High temperatures can disrupt the physiological processes of the crop. These processes include increases in grain sterility and a disruption in the photosynthetic processes, both of which contributes to a decrease in biomass production [64,55]. All of these effects have been linked to a decrease in rice production [61,62]. The temperature variations also correspond to the periodic decrease in rice yields, in some of the major countries such as China, India, Indonesia and Bangladesh [65,57,59]. Similarly, Peng et al. [66] documented several studies which found that production losses were recorded in various locations across China due to heat stress. It has further been recognized [67,68,69] that temperature increases often result in decreased rainfalls, that can result in increased frequencies and intensity of droughts. These conditions further multiply the abiotic stresses which further negatively affects the growth of the crop [69]. The negative impacts of decreased rainfalls and droughts on rice production has been recorded in several studies across various countries [70,71,55].

Increased temperature also affects the rate of respiration at nights, transpiration, and soil health all of which are linked to a subsequent decline in grain reproduction and yields [33]. Gopakumar et al. [72] and Mahdu [50], posited that the variations in temperature and rainfall both influence soil moisture, which lead to the use of advanced drainage and irrigation practices. Countries that have recorded annual declines in production due to temperature variations have begun to implement adaptation measures to improve yield potentials [56]. These include investing in drought and heat tolerant varieties as well as insect and pest-resistant varieties and the development of irrigation infrastructure [73,43,47,74,60].

### 1.2 Irregular rainfall effects on rice production

Rice grows more favourably in well-irrigated, humid conditions. In every stage of cultivation, adequate irrigation is required [50,75]. In many rice producing communities globally, increased frequency of rainfall often leads to flood related losses [76]. On the other hand, reduced frequency of rainfall may contribute to drought induced stress conditions on the crop which reduces production [57]. In the event of droughts, the production is dependent on irrigation systems to obtain a significant yield [59,26]. In the event of early rainfall or monsoon seasons, farmers are likely to face challenges with water availability and soil preparation at the start of the cultivation process [77,50].

Variation in rainfall have contrasting effects on rice production with the production system requiring a finite balance of water over the growing cycle. It is because of this water demand, added to the scale of rice production that is economically feasible, that causes the susceptibility of rice production to changing rainfall patterns. The monsoon period for instance in South Asian countries plays a significant role in cultivation [52]. India, Myanmar and Bangladesh are three of the major rice producers that often have to adjust and manage around the monsoon events. The specific monsoon periods will vary in certain parts of Asia and often determines the rice seasons and varieties that are grown [55]. Early monsoons and flooding often results in multiple stresses on the rice crop and production system [77]. Some of the notable stresses include the delay of transplanting and fertility decrease [33]. In addition to this, the flooded lands caused by the monsoon also decrease the suitability and fertilizer content of the soils, which proves costly for farmers to address. Mahdu [50] summarized that some of the key challenges caused by intense rainfall range from delayed development of the rice crop, costly land preparation activities and increased flood-related pests and rodents on the rice fields.

It was further reported by SeinnSeinn [78] that the farming practices in Myanmar have evolved to reduce the impacts of rainfall variations and to improve yield production. These practices include the implementation of improved methods of water and soil management as well as the use of resilient rice varieties. Bangladeshi farmers integrated climate smart agriculture methods into their rice cultivation practices as an adaptive strategy to deal with the climate variability challenges [77,43].

In the exploration of the literature for this study, majority of the studies empirically explored the impacts of climate change on rice production at the national level [see Refs. [79,80,81,82,83,84]]. There were very limited studies that focused on modelling the impacts of climate change on rice production from a global perspective. By analyzing a global perspective, the empirical evidence can therefore support more collective policy actions in addressing global rice supply issues. In establishing the projected relationships between the climate variables and rice production by modelling, the policy makers in the rice-producing countries will be better equipped to aid farmers in adapting to climate variations and climate change.

This study highlights the importance of modelling spatial and temporal climate data against rice production in an effort to observe the potential impacts of varying temperature and rainfall. It further attempts to act as a baseline study in determining the degree of projected effects of climate variations on global rice outputs and to a larger extent, crop production. Shahid et al. [41] highlighted that the implementation of rice resilient cultivation practices is necessary to maintain high production and yield. However, this can only be done if the national policy makers recognize the limitations of local farmers and how these limitations impact their rice production techniques under changing climatic conditions [61,85,86]. Upon acquisition of this knowledge, further decisions can be made to include developmental programs to aid farmers in adapting to the future impacts of climatic variations on rice production [87].

## 2 Theoretical model

The main underlying objective of this study is to establish an empirical causality between global rice production and variations in precipitation and temperature being experienced globally. As such the model used to establish this causality has the basic functional form:

(1) Y_t,i_ = f(X_1_, X_2_ … X_a_)

Where, Y_t,i_ is the global production of rice over a given time (t) and across (i) a number of countries. From the basic functional form of Eqn (1), the model can then be specified as a linear expression such that:

(2) Y_t,i=_β_0_ + β_a_X_t,i_ + e

Where β_0_ is the constant coefficient, β_a_ represents the vector of coefficients of the exogenous variables in the model, X_t,I_ represent the vector of exogenous variables observed over the time (t) and for the individual countries (i) and (e) represents the error term. Given the literature observed for this study showing the varying effects of rainfall and temperature patterns on the production levels of rice, the econometric model specified is therefore looking at annual rainfall variation and temperatures for all the rice-producing countries in this study so that:

(3) Ln Y_t,i_ = β_0_ + β_1_ LnX_1(t,i)_ + β_2_ LnX_2(t,i,)_ + e

Where, X_1(t,i)_ is the exogenous variable for rainfall across a time period (t) for the individual country (i) and X_2(t,i,)_ is the exogenous variable for temperature across a time period (t) for the individual country (i). The natural logarithm was taken on both sides of the equation to transform the functional form of Eqn (2) into a log-log linear model whereby the coefficient values can be estimated as elasticities. The model coefficients as outlined in Eqn (3) can therefore be interpreted as a percentage change in the dependent variable as a result of a percentage change in the independent variables.

## 3 Methodology

To effectively represent rice production globally, data on production, yield and area harvested were collected for 15 countries all of which have significant rice export markets globally. This list included the top 10 rice-producing countries [88] as well as some smaller producers from regional territories globally. The top ten rice-producing countries and their annual production (in million tonnes) at the time were listed as China (over 148), India (120), Indonesia (34.9), Bangladesh (34.7), Vietnam (27), Thailand (19), Myanmar (12.8), The Philippines (12.1), Pakistan (7.9), and Brazil (7.6) [88].

It was noted that of these top 10 rice producing countries, 9 of them were located in the Asian regions with Brazil being the only top producing country outside of Asia. Taking this into consideration, Guyana and Peru were included with Brazil to aid in further representation for South America. Guyana was also selected as it is one of the Small Island Developing States and a Caribbean country that produces and exports rice. According to FAO [89], Guyana has been working along with the FAO in the collection of data to assist in forecasting crop production to improve planning production for farmers. The USA was then selected as a major producer in North America and it is the 11th top producing country as of 2021. During the data collection process, in 2022, the list of the top ten rice-producing countries was reviewed and updated as necessary. The countries selected for this study are as follows - China, India, Indonesia, Bangladesh, Vietnam, Thailand, Myanmar, The Philippines, Pakistan, Brazil, USA, Japan, Egypt, Peru and Guyana.

Data on each selected country were collected from secondary sources predominantly the FAO stat and World bank open data websites from the February 14, 2022 to the February 28, 2022. Data on rice production (paddy) based on yield (hectograms per hectares), production quantity (tonnes) and area harvested (hectares) for the years 1970–2019 (50 years) were collected from FAO stat open data for each country. This data was sorted and prepared on an excel sheet for later analysis from the March 2, 2022 to the March 5, 2022. The national mean temperature and precipitation data were collected from the World Bank open data website from the March 9, 2022 to the March 11, 2022 for each country. These datasets were filtered based on the 50-year period to be analyzed for this paper and included in the excel data sheet. The compiled data in the excel sheet was then reorganized as a stacked panel dataset with all the countries. This was done to allow for the application of a Panel Regression Model. The regression modelling was done using the Gnu Regression, Econometric Time-series Library (GRETL) software [[90], [91]]. The log values of the variables production, yield, temperature and precipitation were then calculated. A log-log Model was determined to be used in the panel regression model. Here the logged values for both independent and dependent variables were used as inputs in the model. Econometrically, the log-log model provides a neat interpretive value as the estimated coefficients can therefore be interpreted as elasticities [92].

A number of time-series graphs were generated for each country based on the following variables: Production; Yield; Temperature, and Precipitation. For each country, the trend over the time series for production and yield were compared against the trend for temperature and rainfall to identify any relationships between these variables. Additionally, heteroscedasticity corrected weighted least squares panel regression model was implemented with a variable of global rice production from the countries listed in the study as the dependent variable and the varying levels of temperature and rainfall across all the countries in the data set were the independent variables. The weighted panel regression model also adjusts the model for collinearity and auto-correlation issues with the weights based on the per-unit error variances. Diagnostic testing was done looking for cross-sectional dependence to see if any spatial effects needed to be factored in. Mahmood et al. [59] relay that this type of modelling is one of the primary methods of data analysis in crop production and climate variables as it provides tangible estimates in crop production and yield against changes in temperature and rainfall.

## 4 Results and discussion

The findings from the data are presented in two sections. The first section comprises all the time series graphs for the fifteen countries used in this study (see Fig. 1, Fig. 2, Fig. 3, Fig. 4, Fig. 5, Fig. 6, Fig. 7, Fig. 8, Fig. 9, Fig. 10, Fig. 11, Fig. 12, Fig. 13, Fig. 14, Fig. 15). Each figure consists of four graphs (each labelled subsequently a-d) under each figure heading. The second section of the results presents the panel regression model table whereby global rice production was stacked into a panel data set and used as the dependent variable in the model as outlined in Eqn. (3).

**Fig. 1:**
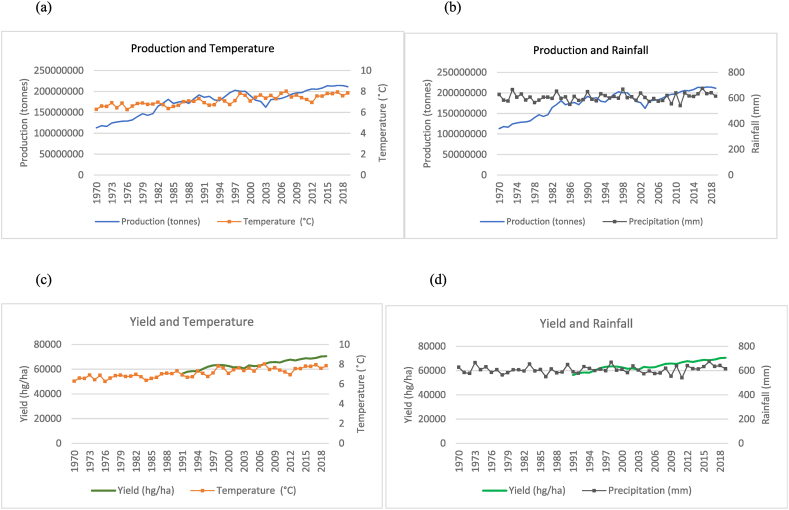
Production and yield against temperature and rainfall for China.

**Fig. 2:**
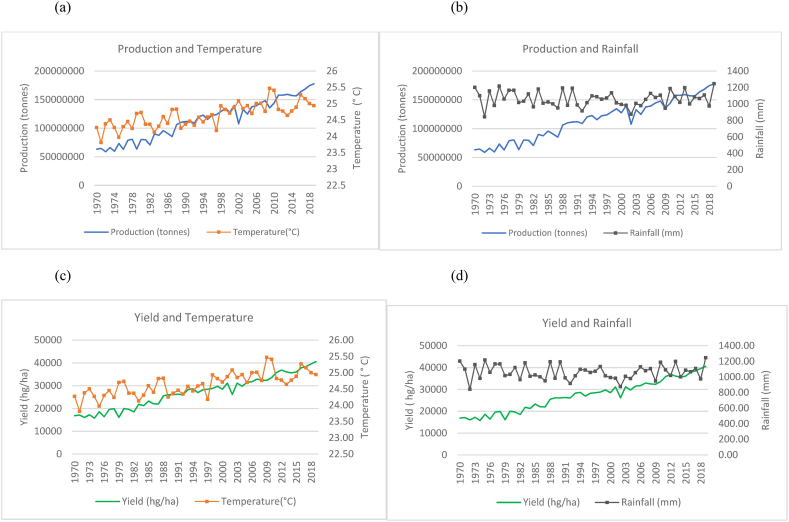
Production and yield against temperature and rainfall for India.

**Fig. 3:**
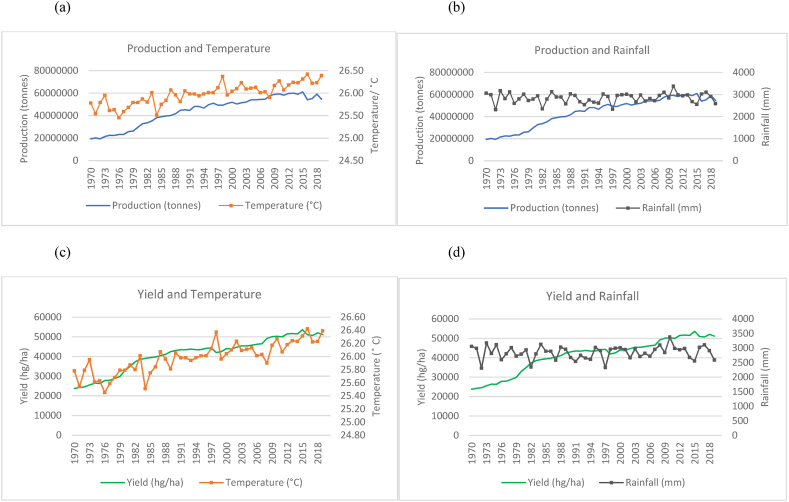
Production and yield against temperature and rainfall for Indonesia.

**Fig. 4:**
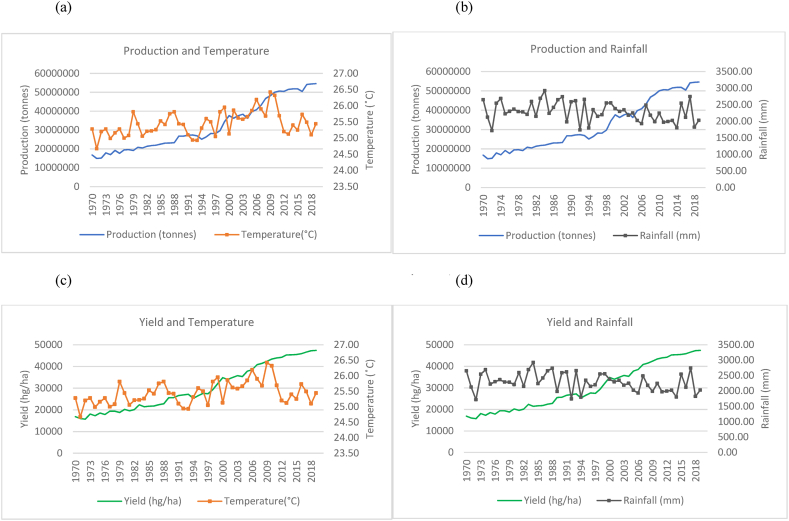
Production and yield against temperature and rainfall for Bangladesh.

**Fig. 5:**
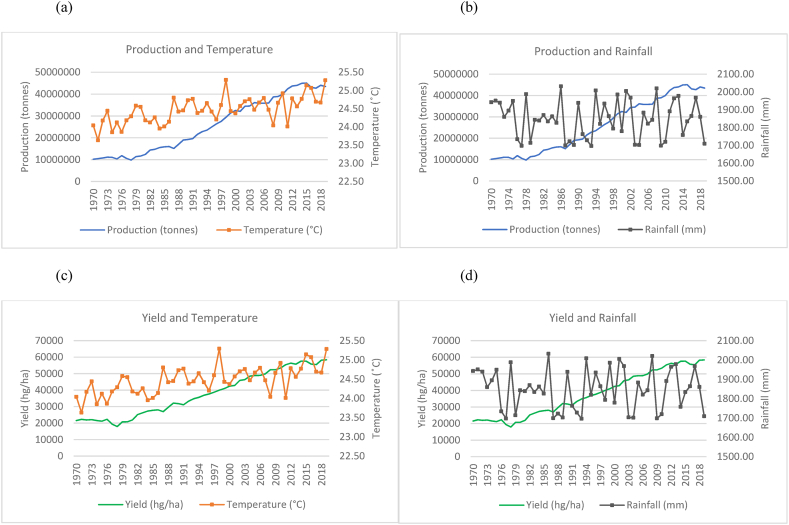
Production and yield against temperature and rainfall for vietnam.

**Fig. 6:**
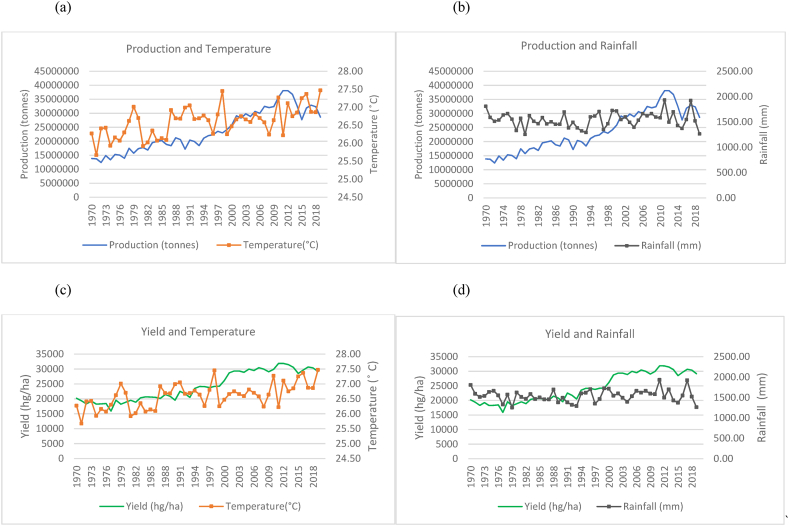
Production and yield against temperature and rainfall for Thailand.

**Fig. 7:**
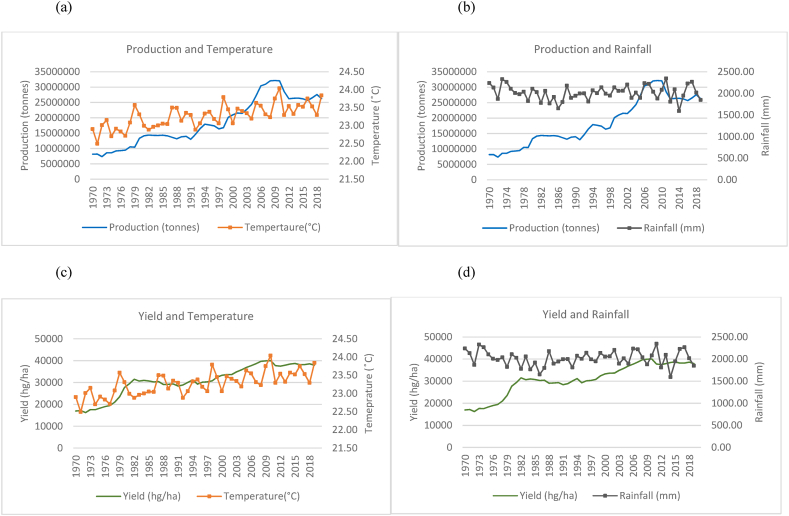
Production and yield against temperature and rainfall for Myanmar.

**Fig. 8:**
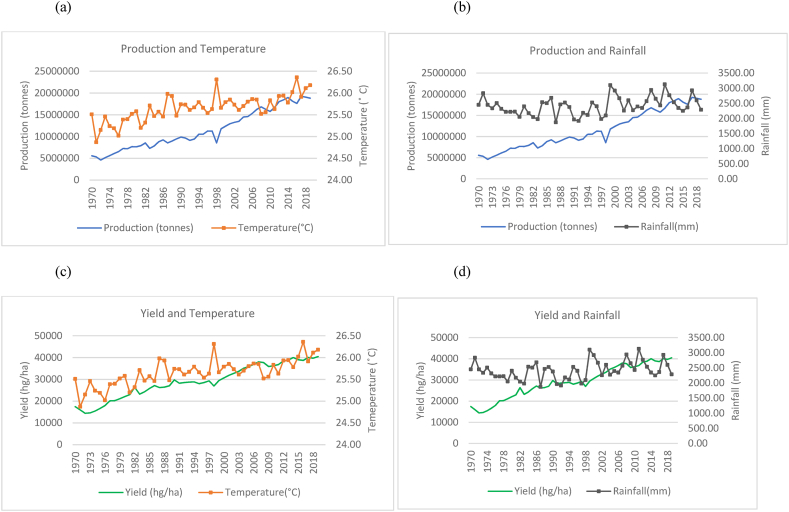
Production and yield against temperature and rainfall for the Philippines.

**Fig. 9:**
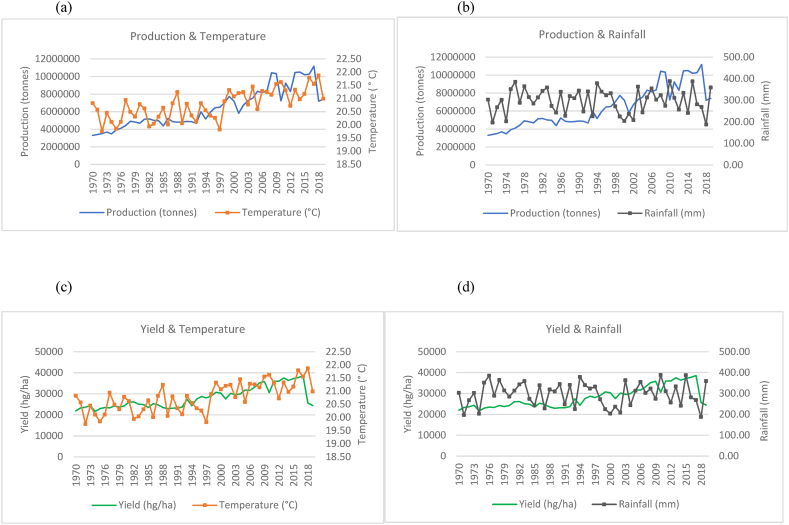
Production and yield against temperature and rainfall for Pakistan.

**Fig. 10:**
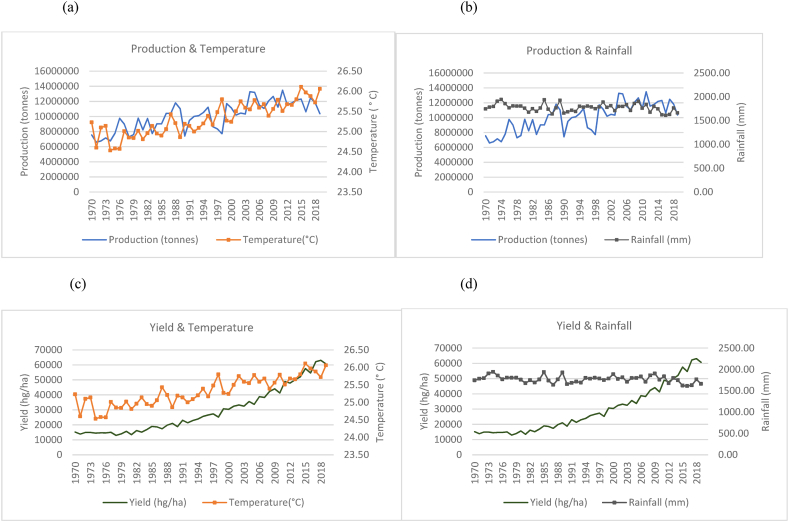
Production and yield against temperature and rainfall for Brazil.

**Fig. 11:**
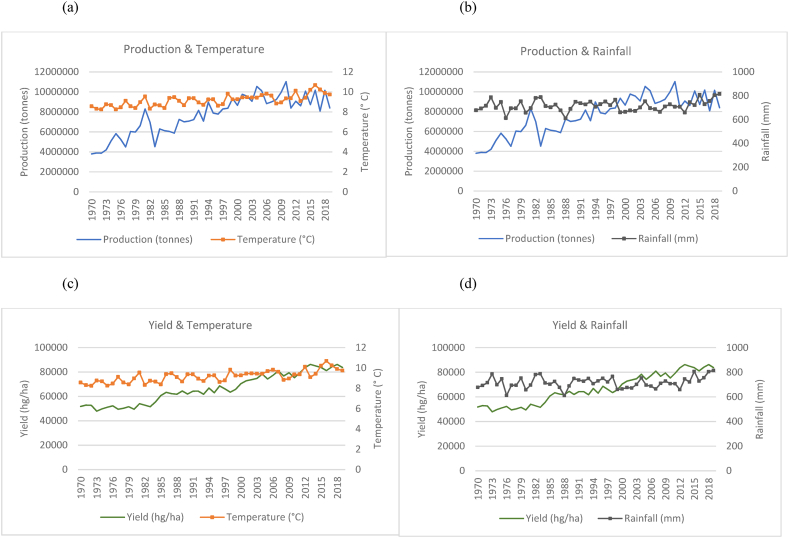
Production and yield against temperature and rainfall for the USA.

**Fig. 12:**
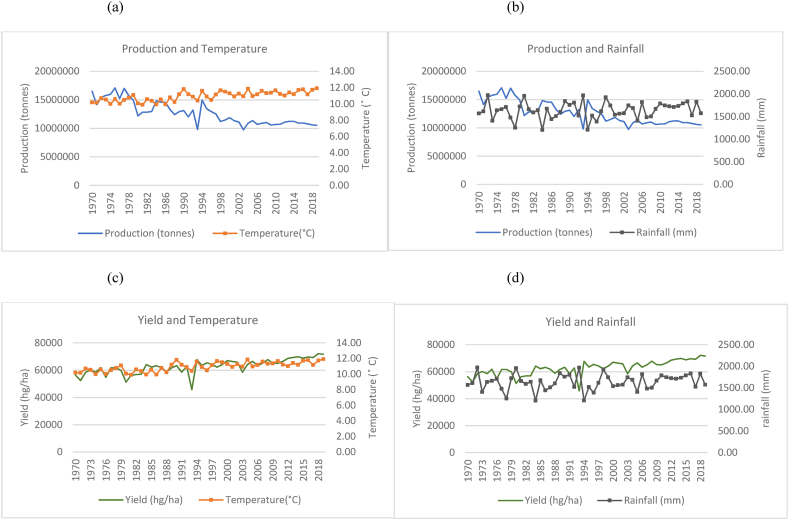
Production and yield against temperature and rainfall for Japan.

**Fig. 13:**
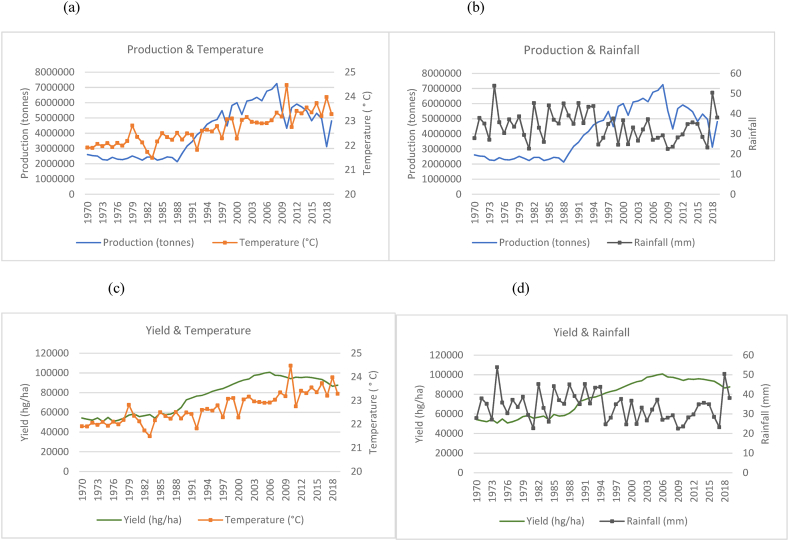
Production and yield against temperature and rainfall for Egypt.

**Fig. 14:**
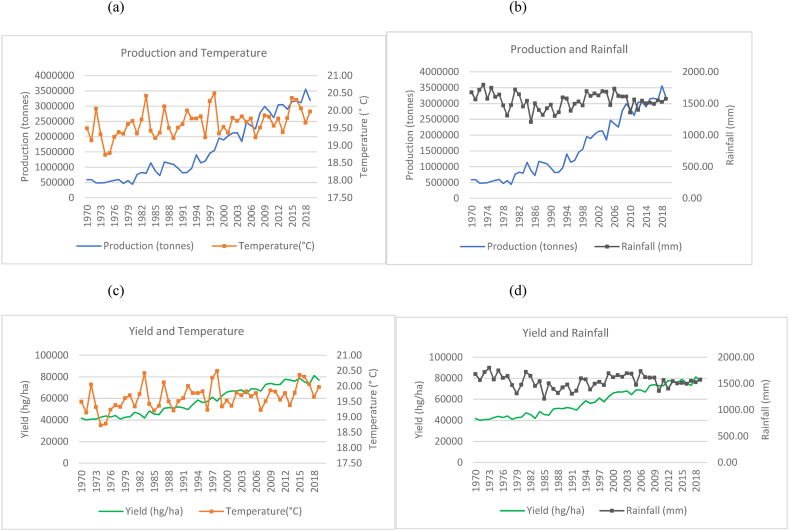
Production and yield against temperature and rainfall for Peru.

**Fig. 15:**
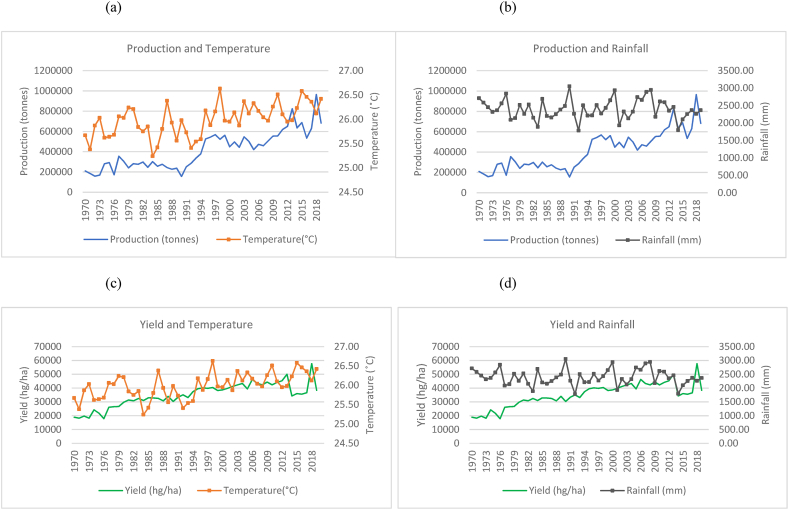
Production and yield against temperature and rainfall for Guyana.

In Fig. 1(a)–(b), it can be seen that the general trend in the production of rice in China has increased from 1970 to 2019 with sporadic fluctuations occurring across the time series. This increase in production coincides with a marginal upward trend in temperature and a relatively horizontal trend in rainfall falling within a range of 500 mm–700 mm annually. In the 50 years observed in the time series, China has doubled its rice production from just over 100, 000, 000 to over 200, 000, 000 tonnes. In Fig. 1(c)–(d), quite similar to the trends observed for production, the country's yield also shows an overall increasing trend despite trends observed for temperatures and rainfalls.

The observed trends for India (as seen in Fig. 2(a)–(b) show a steeper increase in the time series trend for its rice production as compared to China. However, this increase is seen with more evident fluctuations in production when compared to China and Indonesia in Fig. 3. This is seen to occur even with a more evident overall temperature increases and higher rainfall patterns that range between 800 mm and 1300 mm. While India is 2nd to China in terms of the volume of production, the marginal rate of production annually has more than doubled its production from approximately 63, 000, 000 to 178, 000, 000 tonnes. Similarly, Fig. 2(c)–(d) show a general increasing trend in the production of rice per hectare for India despite the fluctuating trends in temperature and rainfall. There were instances where a decline in yield coincides with decreased rainfall. Similarly, dips in production are noticed in years when the country experiences a spike in temperature.

In the case of Indonesia, Fig. 3(a)–(b) show an increasing trend for the production of rice in Indonesia with fewer fluctuations even though the temperature is seen to have an increasing trend over the time series. Rainfall throughout the time series for Indonesia is seen to remain in the range of 2500 mm to 3500 mm. These are higher rainfall levels than that of India and China. In Fig. 3(c)–(d), also show an increase in the yields over the time series despite the increasing trend in temperature and variations in rainfall.

In Fig. 4(a)–(b), Bangladesh has shown an overall increase in rice production over the time series. However, the country showed that the average annual temperature increased over the time series but with large fluctuations year to year. Similarly, Bangladesh's rainfall averages annually remain within a range of over 1500 mm–3000 mm and show a sporadic and cyclical trend moving along a horizontal trajectory. It is noteworthy to identify that a sharp increase in rice production was observed from the year 2000–2019. Even with fluctuating climatic variations, yield (seen in Fig. 4(c)–(d)) in Bangladesh also show a general upward trend, doubling over the time series. In Fig. 5, an increasing trend was observed for temperature variations with consistent rainfall fluctuations in Vietnam. The country however experienced a steady increase in rice production (Fig. 5(a)–(b)) and yield (Fig. 5(c)–(d)) with little patterns in decreased production. It has more than tripled its production in the 50 years from 1970 to 2019. Fig. 6, Thailand is seen to have undergone a general increase in production (Fig. 6(a)–(b)) and yield (Fig. 6(c)–(d)). However, there are multiple occurrences of decreased production across the time series. A temperature increase is also seen across the years and appears to coincide with some production patterns. Rainfall for Thailand has been within a constant range of 1000 mm–2000 mm. The trends between production, yield and temperature for Myanmar as seen in Fig. 7(a)–(c) were observed to be increasing across the time series. It was observed that a general increase in rice production and yield is coinciding with a slight temperature increase in Myanmar. Consistent rainfall is evident from 1970 to 2019 with a relatively horizontal trend with production having an increasing trend (Fig. 7(b)). Nevertheless, it was observed that with instances of peak rainfall, the production per hectare showed a slight decline (Fig. 7(d)).

For the Philippines, a generally positive trend can be seen between temperature, production and yield (Fig. 8(a)–(c)). Even though rainfall displays a constant range against both production and yield (Fig. 8(b)–(d)), the Philippines has experienced a general increase in production and yield over the years which coincides with the slight temperature increase. Nevertheless, Fig. 8(b)–(d) show that with peak rainfalls, the yield experiences a slight decline for various years which is similar to Myanmar. For Pakistan, a general increase can be seen in both production and yield against the subtle temperature increase and consistent rainfall patterns (Fig. 9a–d). Despite the increase, when examined closely, it can be observed that temperature and rice production share a marginal mutually fluctuating trend. As temperature peaks and declines, rice production and yield are seen to follow (Fig. 9(a)–(c)). As for Brazil, it can be seen that both temperature and yield share a positive increasing trend (Fig. 10(a)– (c)). While there is an overall increase in both rice production and yield, the fluctuations with both variables match that of the temperature patterns across the time series. Rainfall on the other hand has displayed a consistent pattern in Brazil from 1970 to 2019. These patterns do not coincide with the increased rice production and yield trends (Fig. 10(b)–(d)).

In Fig. 11(a), the annual mean temperature for the US shows an increase over the 50 years from an average of 8 °C to just over 10 °C. However, rainfall variations remain between the ranges of 600 mm–800 mm as such, illustrating a constant trend (Fig. 11(b)). Nevertheless, rice production and yield in the US show an overall increase with a relative frequency of fluctuations (Fig. 11(a)–(c)). Production, however, shows a steeper increase as opposed to yield. As seen in Fig. 11(b)–(d), production and yield continue to gradually increase from the year 1997. For Japan, in Fig. 12(a), there is a distinct inversely proportional relationship between temperature and rice production observed. Fig. 12(b) shows that rainfall remains fairly consistent within a range of 1200 mm–2000 mm. However, the amount of rice harvested per hectare directly coincides with the temperature pattern with minor differences (Fig. 12(c)). It can be seen that even though rice production has declined, the amount harvested per hectare gradually increases even with temperature and rainfall fluctuations (Fig. 12(c)–(d)). Egypt has experienced a gradual temperature increase and a slight decrease in rainfall in the 50 years with sudden spikes in both variables (Fig. 13(a)– (b)). In spite of this, rice production and yield have increased (Fig. 13(a)–(c)). Upon making a more critical observation, between the years 1998–2018, when rainfall appears to be in the lowest range, the country experiences a spike in both production and yield. On a general basis, the quantities of temperature, production and yield share a positive relationship according to the trends. Production and yield have an inverse relationship with rainfall based on the trends observed in the time series. With distinct rainfall patterns over the years, both production and yield have increased with the decrease in rainfall (Fig. 13(b)–(d)). It can be seen that while rice production in Peru fluctuates with small peaks, it has increased substantially in its production over the time series (Fig. 14(a)) despite changing temperatures and a general decline in rainfall (Fig. 14(a)–(b)). As compared to the increase in yield per hectare with a more gradual trend (Fig. 14(c)–(d)), total rice production has seen a steeper increase implying the country is expanding its rice production areas.

In the case of Guyana, Fig. 15(a) shows that the country has experienced a general increase in rice production over the time series. It is also evident that the country has seen a slight increase in temperature while its rainfall patterns remain consistent in the range of 2000 mm–3000 mm (Fig. 15(b)). The country's yield has also increased despite the varying temperature and rainfall patterns (Fig. 15(c) and (d)). There are instances across the time series where the decrease in rainfall results in a higher amount of rice harvested per hectare (Fig. 15(d)).

Based on the time series trends observed for the 15 countries, it was noted that the vast majority of the countries (93% of the sample) showed an increasing trend in their rice production levels and the productivity of rice fields with some countries tripling their production levels over the 50-year period. All of the data showed increasing trends in the average annual temperatures for all the countries in the study and the majority of the countries showed relatively consistent precipitation volumes. Some countries such as Egypt showed a marginal decrease in their average annual rainfall over the time series but this did not impact the rice production levels for the country. However, it can be postulated that for some countries, a decline in yield is linked to the highest rainfall volumes experienced. This observation was also highlighted by several recent studies [see Refs. [93,94,95]].

The temperature variations experienced in these countries ranged between 0.5 and 2 °C from the years 1970–2019. These variations are detected by taking the differences between lowest and the maximum temperatures from the countries. In alignment with this finding, Chou et al. [96] and the IPCC [1] shown that relative to pre-industrialization, by 2005, the global temperature was an average 0.6 °C warmer. In spite of the temperature variations, the increasing trends suggest that the temperatures were generally favorable to allow for the continued increase in rice production among the countries. Nevertheless, multiple studies have shown that if the temperature variations decrease below the annual minimum temperature or exceed the maximum temperature ranges of a region, it is likely that rice production will be negatively impacted [59,26]. The time series trends observed for temperature and rainfall are consistent with the climate variations model in the literature [see Refs. [97,98]].

Even with the expected increases in temperature and fluctuations in rainfall, the increase in production and productivity essentially implies that global rice production is not significantly impacted by climatic variations on the macro scale at this point. The study did not look at production at the community level across countries which may show a different outlook when it comes to the localized impacts on rice production caused by climate variabilities. Nevertheless, literature explored, indicates that regional climate variations share a strong relationship with increased and more intensified occurrences of extreme climate events, such as droughts, heat waves, storms, floods, etc. [see Refs. [96,99,100,101]].

The increasing trends in rice production and the productivity observed in the data can be attributed to several factors such as improved agronomy practices, the incorporation of more climate-smart technology in rice production and the growing demand for rice globally which offers economic incentives for the rice producers to expand production. This finding corroborates with the literature as countries have well documented the successes of their agronomy strategies in strengthening their rice production systems to withstand adverse climatic conditions [102,103].

Even though the majority of the data across the time series showed an upward trend in rice production, disaggregating the data showed that annual variations in production and the productivity that coincide with fluctuations in temperature and rainfall. Some countries showed a sharp increase in production over time. Countries such as Thailand, Pakistan, Egypt, Brazil, USA, Peru and Guyana showed more distinct increasing trends in rice production but these countries showed more significant annual decreases in rice production and yield that coincide with temperature and rainfall variations in specific years. Japan is the lone country that exhibited a decline in rice production against increased temperature and rainfall. Based on the evidence seen from the other countries, the reasoning for the decline in rice production in Japan is most likely not due to climatic variations but as a likely result of the economic transformations made by the country [see Refs. [104,105,106]]. Japan's economy has transformed into one that is technology-driven and service based with agriculture in general steadily declining over time [see Refs. [107,108]].

### 4.1 Panel regression model

Observing the global trends of rice production against temperature increase and rainfall patterns showed that rice production overall is not impacted presently. To further explore the empirical relationship between these climatic variables and rice production, a weighted least squares (WLS) panel regression model was adopted by aggregating global rice production data and regressing it against rainfall and temperature averages for the respective countries used in this study. The model was corrected for heteroscedasticity, autocorrelation and multi-collinearity by using the error variances as weights in the regression model. A Pesaran Cross Dependence test was conducted and showed no spatial dependence in the panel data. The model findings align with the literature on the anticipated impacts of climate variations and climate change on rice production [see Refs. [109,110,100,111]].

Table 1 showed that all the coefficients for the variables of rainfall and temperature were found to be significant with *P*-values less than 1%. The model showed a positive coefficient for rainfall and a negative coefficient for temperature. A percentage increase in rainfall will cause a percentage increase in rice production and vice versa. Whereas, if there is a percentage increase in temperature in the future then there will be a percentage decrease in rice production. This finding from the model coincides with the results of several studies that indicated a likely decline in rice production in the event of increased temperatures [see Refs. [112,87,99,86,100]]. Recent studies have shown that the rice producing countries have started to explore and implement adaptive and mitigative strategies to maintain positive rice production and hence food security [see Refs. [62,113,85,41]].

**Table 1:** Weighted least squares panel regression model on global rice production.

Variable	Coefficient	Std. Error
Constant	2.79432***	0.131304
Ln Rainfall	0.120744***	0.0147039
Ln Temperature	−0.243386***	0.0312896
Ln Area	0.892282***	0.00875511

Note: ***, **,* represents statistical significance at 1%, 5% and 10% respectively. Measures of goodness of fit include the R-square value (0.936086) and adjusted R-square value (0.935829). The model has a statistically significant F-value of 1%.

The regression model diagnostics showed an R-square and an adjusted R-square value of 0.93 which indicated that the explanatory variables in the model account for 93% of the variation in rice production. This indicates a well-defined model and the strong relationship between the climatic variables of temperature and precipitation with rice production. This finding correlates to the literature explored that temperature and rainfall changes are linked to rice production [see Refs. [61,114,26,76]]. Despite this, in addition to changes in temperature and rainfall, several other physiological factors such as soil moisture and fertilizer use, are known to affect the outputs of rice production [115,7]. Since the model only used datasets on climate variations, further research can be done by using datasets of the physiological factors to determine how they can alleviate the negative effects of climate variations on rice production.

Additionally, since the datasets used in this study were taken on a national level, further critical analyses can be done using specific datasets from the rice producing areas in the countries that experience distinct spatial temperature and rainfall differences, such as the USA, China, India and Brazil. Additional research can be done to determine whether the socio-economic factors influence global rice production. It is further recommended that this research and other studies on rice production and climate variations be taken into consideration to aid in the preparation and mitigation of the impacts that climate variations will have on the regional and global economies.

## 5 Conclusion

An empirical analysis was conducted looking at global rice production and its relationship with the climatic variations of temperature and rainfall experienced globally. The analysis was conducted in two phases. The first phase was a time series trend analysis to observe similarities in the trends for rice production, temperature and rainfall and the second phase was to establish causality between rice production and temperature and rainfall using regression modelling. Interestingly, the time series trends showed that the majority of the major rice-producing countries have increased their volume of production despite the increase in temperature and variations in rainfall. This illustrates that the aggregated production of rice remains unaffected by the current climatic conditions as production and yield continue to increase. The increasing volumes of rice production are an indication of the continued importance and demand for rice as a staple.

The panel regression model showed that rice production is significantly impacted by precipitation and temperature changes and if temperatures continue to increase, production levels are expected to eventually decrease. As temperature and rainfall continue to change worldwide, the global rice production supply will start to be impacted. With the demand for rice expected to increase by 25% by 2030, it is recommended that the rice-producing countries invest in more adaptation policies and strategies specially to deal with increasing temperatures in order to meet this global demand.

The study's findings further corroborate with the literature, but it was the first attempt to implement a global model for rice production whereby the literature predominantly focuses on community or national impacts. Essentially, the global outlook for rice has an uncertain future as the effects of climate change in the form of climate variations become more notable.

## Author contribution statement

Masha Joseph and Stephan Moonsammy: Conceived and designed the experiments; Performed the experiments; Analyzed and interpreted the data; wrote the paper.

Harold Davis, Devin Warner, Ashley Adams and Timothy Oyedotun: Contributed reagents, materials, analysis tools or data; wrote the paper.

## Data availability statement

Data will be made available on request.

## Additional information

No additional information is available for this paper.

## Declaration of competing interest

The authors declare that they have no known competing financial interests or personal relationships that could have appeared to influence the work reported in this paper.

## References

[1] Masson-Delmotte V., Zhai P., Pörtner H.-O., Roberts D., Skea J., Shukla P.R., Pirani A., Moufouma-Okia W., Péan C., Pidcock R., Connors S., Matthews J.B.R., Chen Y., Zhou X., Gomis M.I., Lonnoy E., Maycock T., Tignor M., Waterfield T., IPCC (2018). Global Warming of 1.5°C. An IPCC Special Report on the Impacts of Global Warming of 1.5°C above Pre-industrial Levels and Related Global Greenhouse Gas Emission Pathways, in the Context of Strengthening the Global Response to the Threat of Climate Change, Sustainable Development, and Efforts to Eradicate Poverty.

[2] Aryal J.P., Sapkota T.B., Khurana R., Khatri-Chhetri A., Rahut D.B., Jat M.L. (2020). Climate change and agriculture in South Asia: adaptation options in smallholder production systems. Environ. Dev. Sustain..

[3] Del Buono D. (2021). Can biostimulants be used to mitigate the effect of anthropogenic climate change on agriculture? It is time to respond. Sci. Total Environ..

[4] Masson-Delmotte V., Zhai P., Pörtner H.-O., Roberts D., Skea J., Shukla P.R., Pirani A., Moufouma-Okia W., Péan C., Pidcock R., Connors S., Matthews J.B.R., Chen Y., Zhou X., Gomis M.I., Lonnoy E., Maycock T., Tignor M., Waterfield T., Intergovernmental Panel on Climate Change (2018). https://www.ipcc.ch/sr15/chapter/spm/.

[5] Karimi V., Karami E., Keshavarz M. (2018). Climate change and agriculture: impacts and adaptive responses in Iran. J. Integr. Agric..

[6] Wu F., Wang Y., Liu Y., Liu Y., Zhang Y. (2021). Simulated responses of global rice trade to variations in yield under climate change: evidence from main rice-producing countries. J. Clean. Prod..

[7] Xu G., Lu D., Hezheng W., Li Y. (2018). Morphological and physiological traits of rice roots and their relationships to yield and nitrogen utilization as influenced by irrigation regime and nitrogen rate. Agric. Water Manag..

[8] Zhang L., Zhang Z., Tao F., Luo Y., Zhang J., Cao J. (2022). Adapting to climate change precisely through cultivars renewal for rice production across China: when, where, and what cultivars will be required?. Agric. For. Meteorol..

[9] Shukla P.R., Skea J., Calvo Buendia E., Masson-Delmotte V., Pörtner H.-O., Roberts D.C., Zhai P., Slade R., Connors S., van Diemen P., Ferrat M., Haughey E., Luz S., Neogi S., Pathak M., Petzold J., Portugal Pereira J., Vyas P., Huntley E., Malley J., Intergovernmental Panel on Climate Change (2019). Climate Change And Land: an IPCC Special Report on Climate Change, Desertification, Land Degradation, Sustainable Land Management, Food Security, and Greenhouse Gas Fluxes in Terrestrial Ecosystems.

[10] Arora N.K. (2019). Impact of climate change on agriculture production and its sustainable solutions. Environ. Sustain..

[11] Bradshaw C., Kay G., Davie J., Cottrell A., Bacon J., Cosse A., Dunstone N., Jennings S., Challinor A., Chapman S., Birch C., Sallu S.M., King R., Macdiarmid J., Pope E. (2022). Unprecedented climate extremes in South Africa and implications for maize production. Environ. Res. Lett..

[12] Lobell D.B., Burke M.B. (2008). Why are agricultural impacts of climate change so uncertain? The importance of temperature relative to precipitation. Environ. Res. Lett..

[13] Nielsen-Gammon J.W., Banner J.L., Cook B.I., Tremaine D.M., Wong C.I., Mace R.E., Gao H., Yang Z., Gonzalez M.F., Hoffpauir R., Gooch T., Kloesel K. (2020). Unprecedented drought challenges for Texas water resources in a changing climate: what do researchers and stakeholders need to know?. Earth's Future.

[14] De Haen H. (2008). Food Security Strategies: building Resilience against Natural Disasters Stratégies de sécurité alimentaire : améliorer la résistance aux catastrophes naturelles Strategien für die Sicherung der Ernährung: stärkung der Widerstandsfähigkeit gegen Naturkatas. EuroChoices.

[15] Kaur S., Kaur N., Yousuf A., Singh J., Sandhu P.S. (2022).

[16] Kogo B.K., Kumar L., Koech R. (2021). Climate change and variability in Kenya: a review of impacts on agriculture and food security. Environment. Development and Sustainability.

[17] Rwanyiziri G., Uwiragiye A., Tuyishimire J., Mugabowindekwe M., Mutabazi A., Hategekimana S., Mugisha J. (2019). Assessing the impact of climate change and variability on wetland maize production and the implication on food security in the highlands and central plateaus of Rwanda. Ghana J. Geog..

[18] Wheeler T., von Braun J. (2013). Climate change impacts on global food security. Science.

[19] Wu X., Jiang D. (2022). Probabilistic impacts of compound dry and hot events on global gross primary production. Environ. Res. Lett..

[20] Zimmerman A., Benda J., Webber H., Jafari Y. (2018). https://www.fao.org/3/CA2370EN/ca2370en.pdf.

[21] Poutanen K.S., Kårlund A.O., Gómez-Gallego C., Johansson D.P., Scheers N.M., Marklinder I.M., Eriksen A.K., Silventoinen P.C., Nordlund E., Sozer N., Hanhineva K.J., Kolehmainen M., Landberg R. (2022). Grains – a major source of sustainable protein for health. Nutr. Rev..

[22] Awika J.M. (2011). Major cereal grains production and use around the World. ACS Symp. Ser..

[23] Nyiraguhirwa S., Grana Z., Ouabbou H., Iraqi D., Ibriz M., Mamidi S., Udupa S.M. (2022). A genome-wide association study identifying single-nucleotide polymorphisms for iron and zinc biofortification in a worldwide barley collection. Plants.

[24] Ray D.K., Gerber J.S., MacDonald G.K., West P.C. (2015). Climate variation explains a third of global crop yield variability. Nat. Commun..

[25] Dikitanan R.C., Pede V.O., Rejesus R.M., Bhandari H., Alam G.M., Andrade R.S. (2022). Assessing returns to research investments in rice varietal development: evidence from the Philippines and Bangladesh. Global Food Secur..

[26] Rahman M.A., Kang S., Nagabhatla N., Macnee R. (2017). Impacts of temperature and rainfall variation on rice productivity in major ecosystems of Bangladesh. Agric. Food Secur..

[27] Urom C., Guesmi K., Abid I., Enwo-Irem I.N. (2022). Co-inventions, uncertainties and global food security. Environ. Econ. Pol. Stud..

[28] Bvenura C., Kambizi L. (2022). Future grain crops. Future Foods.

[29] Firdaus R.B.R., Leong Tan M., Rahmat S.R., Senevi Gunaratne M. (2020). Paddy, rice and food security in Malaysia: a review of climate change impacts. Cogent Soc. Sci..

[30] Mukherjee S., Nandi R., Kundu A., Bandyopadhyay P.K., Nalia A., Ghatak P., Nath R. (2022). Soil water stress and physiological responses of chickpea (Cicer arietinum L.) subject to tillage and irrigation management in lower Gangetic plain. Agric. Water Manag..

[31] Singh S., Awais M. (2019). Climate variability and rice production in North India: a review. Econ. Aff..

[32] Wang X., Qiang W., Niu S., Growe A., Yan S., Tian N. (2022). Multi-Scenario simulation analysis of grain production and demand in China during the peak population period. Foods.

[33] Wassmann R., Dobermann A. (2007). Climate change adaptation through rice production in regions with high poverty levels. https://citeseerx.ist.psu.edu/viewdoc/download?doi=10.1.1.524.9007&rep=rep1&type=pdf.

[34] Wei X., Zhang Z., Wang P., Tao F. (2016). Recent patterns of production for the main cereal grains: implications for food security in China. Reg. Environ. Change.

[35] International Rice Research Institute (2019). Annual report 2019- race for impact. https://www.irri.org/resources-and-tools/publications.

[36] Naik P.L., Kotecha M., Nathani S., Rathore B.S. (2022). Rice-A review of nutritional and medicinal aspect mentioned in ayurveda. South Asian Res. J. Pharm. Sci..

[37] Muthayya S., Sugimoto J.D., Montgomery S., Maberly G.F. (2014). An overview of global rice production, supply, trade, and consumption. Ann. N. Y. Acad. Sci..

[38] Chaudhari P.R., Tamrakar N., Singh L., Tandon A., Sharma D. (2018). Rice nutritional and medicinal properties: a. J. Pharmacogn. Phytochem..

[39] Khan N., Chowdhary P., Ahmad A., Giri B.S., Chaturvedi P. (2020). Hydrothermal liquefaction of rice husk and cow dung in Mixed-Bed-Rotating Pyrolyzer and application of biochar for dye removal. Bioresour. Technol..

[40] Morin-Crini N., Lichtfouse E., Torri G., Crini G. (2019). Applications of chitosan in food, pharmaceuticals, medicine, cosmetics, agriculture, textiles, pulp and paper, biotechnology, and environmental chemistry. Environ. Chem. Lett..

[41] Shahid M., Munda S., Khanam R., Chatterjee D., Kumar U., Satapathy B., Mohanty S., Bhaduri D., Tripathi R., Nayak P.K., Nayak A.K. (2021). Climate resilient rice production system: natural resources management approach. Oryza-An Int. J. Rice.

[42] Surendran U., Raja P., Jayakumar M., Subramoniam S.R. (2021). Use of efficient water saving techniques for production of rice in India under climate change scenario: a critical review. J. Clean. Prod..

[43] IPCC (2013). https://books.google.gy/books?hl=en&lr=&id=o4gaBQAAQBAJ&oi=fnd&pg=PR1&ots=Whpz8PBqRj&sig=cJT2Xu_sXmLjsBPGRlVHFgu4b6U&redir_esc=y#v=onepage&q&f=false.

[44] Persaud M., Persaud R., Gobind N., Khan A., Corredor E. (2022). Genotype by environment interactions of grain yield performance and lodging incidence in advance breeding lines of rice across environments in Guyana. https://journalissues.org/ijapr/wp-content/uploads/sites/5/2022/05/Persaud-et-al-.pdf.

[45] Delerce S., Dorado H., Grillon A., Rebolledo M.C., Prager S.D., Patiño V.H., Garcés Varón G., Jiménez D. (2016). Assessing weather-yield relationships in rice at local scale using data mining approaches. PLoS One.

[46] Luo W., Chen M., Kang Y., Li W., Li D., Cui Y., Khan S., Luo Y. (2022). Analysis of crop water requirements and irrigation demands for rice: implications for increasing effective rainfall. Agric. Water Manag..

[47] Korres N.E., Norsworthy J.K., Burgos N.R., Oosterhuis D.M. (2017). Temperature and drought impacts on rice production: an agronomic perspective regarding short-and long-term adaptation measures. Water Res. Rural Dev..

[48] Lyman N.B., Jagadish K.S.V., Nalley L.L., Dixon B.L., Siebenmorgen T. (2013). Neglecting rice milling yield and quality underestimates economic losses from High-Temperature stress. PLoS One.

[49] Welch J.R., Vincent J.R., Auffhammer M., Moya P.F., Dobermann A., Dawe D. (2010). Rice yields in tropical/subtropical Asia exhibit large but opposing sensitivities to minimum and maximum temperatures. Proc. Natl. Acad. Sci. USA.

[50] Mahdu O. (2019). https://vtechworks.lib.vt.edu/handle/10919/89087.

[51] Ayinde O.E., Ojehomon V.E.T., Daramola F.S., Falaki A.A. (2013). Evaluation of the effects of climate change on rice production in Niger State, Nigeria. Ethiopian J. Environ. Stud. Manag..

[52] Adib M.N.M., Harun S., Rowshon M.K. (2022). Long-term rainfall projection based on CMIP6 scenarios for Kurau River Basin of rice-growing irrigation scheme, Malaysia. SN Appl. Sci..

[53] Chen M., Cui Y., Wang X., Xie H., Liu F., Luo T., Zheng S., Luo Y. (2021). A reinforcement learning approach to irrigation decision-making for rice using weather forecasts. Agric. Water Manag..

[54] Ding Y., Wang W., Zhuang Q., Luo Y. (2020). Adaptation of paddy rice in China to climate change: the effects of shifting sowing date on yield and irrigation water requirement. Agric. Water Manag..

[55] Shelley I.J., Takahashi-Nosaka M., Kano-Nakata M., Haque M.S., Inukai Y. (2016). Rice cultivation in Bangladesh: present scenario, problems, and prospects. J. Int. Cooper. Agric. Dev..

[56] Tan B.T., Fam P.S., Firdaus R.R., Tan M.L., Gunaratne M.S. (2021). Impact of climate change on rice yield in Malaysia: a panel data analysis. Agriculture.

[57] Huang Y., Zhang W., Yu Y., Sun W., Sun W., Chen J. (2009). A primary assessment of climate change impact on rice production in China. IOP Conf. Ser. Earth Environ. Sci..

[58] Zhang H., Zhou G., Liu D.L., Wang B., Xiao D., He L. (2019). Climate-associated rice yield change in the Northeast China Plain: a simulation analysis based on CMIP5 multi-model ensemble projection. Sci. Total Environ..

[59] Mahmood N., Ahmad B., Hassan S., Bakhsh K. (2012). Impact of temperature AND precipitation on rice productivity in rice-wheat cropping system of Punjab province. J. Anim. Plant Sci.

[60] Waltthal C.L., Hadfield J., Backlund P., Lengnick L., Marshall E., Walch M., Adkins S., Aillery M., Ainsworth E.A., Ammann C., Anderson C.J., Bartomeus I., Baumgard L.H., Booker F., Bradley B., Blumenthal D.M., Bunce J., Burkey K., Dabney S.M., Ziska L.H. (2013).

[61] Chung N.T., Jintrawet A., Promburom P. (2015). Impacts of seasonal climate variability on rice production in the central highlands of Vietnam. Agric. Agric. Sci. Procedia.

[62] Costa A., Thanarajoo S.S., Sivapragasam A. (2018). https://hdl.handle.net/10568/97537.

[63] Shahzad A., Ullah S., Dar A.A., Sardar M.F., Mehmood T., Tufail M.A., Shakoor A., Haris M. (2021). Nexus on climate change: agriculture and possible solution to cope future climate change stresses. Environ. Sci. Pollut. Control Ser..

[64] Kinose Y., Masutomi Y., Shiotsu F., Hayashi K., Ogawada D., Gomez-Garcia, Matsumura A., Takahashi K., Fukushi K. (2020). Impact assessment of climate change on the major rice cultivar Ciherang in Indonesia. J. Agric. Meteorol..

[65] Caruso R., Petrarca I., Ricciuti R. (2016). Climate change, rice crops, and violence. J. Peace Res..

[66] Peng S., Tang Q., Zou Y. (2009). Current status and challenges of rice production in China. Plant Prod. Sci..

[67] Da Costa M.C., Ramegowda Y., Ramegowda V., Karaba N.N., Sreeman S.M., Udayakumar M. (2021). Combined drought and heat stress in rice: responses, phenotyping and strategies to improve tolerance. Rice Sci..

[68] Mukamuhirwa A., Hovmalm H.P., Bolinsson H., Ortiz R., Nyamangyoku O., Johansson E. (2019). Concurrent drought and temperature stress in rice—a possible result of the predicted climate change: effects on yield attributes, eating characteristics, and health promoting compounds. Int. J. Environ. Res. Publ. Health.

[69] Piveta L.B., Burgos N.R., Noldin J.A., Viana V.E., De Oliveira C.M., Lamego F.P., De Avila L.A. (2020). Molecular and physiological responses of rice and weedy rice to heat and drought stress. Agriculture.

[70] Bernier J., Atlin G.N., Serraj R., Kumar A., Spaner D. (2008). Breeding upland rice for drought resistance. J. Sci. Food Agric..

[71] Pandey S., Behura D.D., Villano R., Naik D. (2000). Economic cost of drought and farmers' coping mechanisms: a study of rainfed rice systems in Eastern India (No. 2169-2019-1610). https://ageconsearch.umn.edu/record/287599/files/Pandey.pdf.

[72] Gopakumar C.S., Prasada Rao G.S.L.H.V., Ram Mohan H.S. (2011). https://dyuthi.cusat.ac.in/xmlui/bitstream/handle/purl/2940/Dyuthi-T0931.pdf;sequence=1.

[73] Birthal P.S., Negi D.S., Khan M.T., Agarwal S. (2015). Is Indian agriculture becoming resilient to droughts? Evidence from rice production systems. Food Pol..

[74] Wang Y., Huang J., Wang J., Findlay C. (2018). Mitigating rice production risks from drought through improving irrigation infrastructure and management in China. Aust. J. Agric. Resour. Econ..

[75] Mondol M.A.H., Zhu X., Dunkerley D., Henley B.J. (2022). Changing occurrence of crop water surplus or deficit and the impact of irrigation: an analysis highlighting consequences for rice production in Bangladesh. Agric. Water Manag..

[76] Rokonuzzaman M., Rahman M.M., Yeasmin M., Islam M.S. (2018). Relationship between precipitation and rice production in Rangpur district. Progressive Agriculture.

[77] Ali M.Y., Hossain M.E. (2019). “Profiling climate smart agriculture for southern coastal region of Bangladesh and its impact on productivity, adaptation and mitigation”. EC Agriculture.

[78] SeinnSeinn M.U., Ahmad M.M., Thapa J.P., Shrestha R.P. (2015). Farmers' adaptation to rainfall variability and salinity through agronomic practices in lower ayeyarwady delta, Myanmar. J. Earth Sci. Climatic Change.

[79] Abbas S., Kousar S., Shirazi S.A., Yaseen M., Latif Y. (2022). Illuminating empirical evidence of climate change: impacts on rice production in the Punjab regions. Pakistan. Agric. Res..

[80] Ansari A., Lin Y.P., Lur H.S. (2021). Evaluating and adapting climate change impacts on rice production in Indonesia: a case study of the Keduang Subwatershed. Central Java. Environ..

[81] Arunrat N., Pumijumnong N. (2015). The preliminary study of climate change impact on rice production and economic in Thailand. Asian Soc. Sci..

[82] Lv Z., Zhu Y., Liu X., Ye H., Tian Y., Li F. (2018). Climate change impacts on regional rice production in China. Climatic Change.

[83] Rimi R.H., Rahman S.H., Karmakar S., Hussain S.G. (2009). Trend analysis of climate change and investigation on its probable impacts on rice production at Satkhira, Bangladesh. Pakistan J. Meteorol..

[84] Zubair L., Nissanka S.P., Weerakoon W.M.W., Herath D.I., Karunaratne A.S., Prabodha A.S.M., McDermid S. (2015).

[85] Pickson R.B., He G., Boateng E. (2022). The impacts of climate change and smallholder farmers' adaptive capacities on rice production in Chengdu, China: macro-micro analysis. Environ. Res. Commun..

[86] Raza A., Razzaq A., Mehmood S., Zou X., Zhang X., Lv Y., Xu J. (2019). Impact of climate change on crops adaptation and strategies to tackle its outcome: a review. Plants.

[87] Baig I.A., Chandio A.A., Ozturk I., Kumar P., Khan Z.A., Salam M. (2022). Assessing the long- and short-run asymmetrical effects of climate change on rice production: empirical evidence from India. Environ. Sci. Pollut. Control Ser..

[88] USDA (2022). Rice sector at a glance. https://www.ers.usda.gov/topics/crops/rice/rice-sector-at-a-glance/.

[89] Food and Agriculture Organization (2014). https://sustainabledevelopment.un.org/content/documents/2246fao%20in%20sids.pdf.

[90] Baiocchi G., Distaso W. (2003). GRETL: econometric software for the GNU generation. J. Appl. Econom..

[91] Rosenblad A. (2008). gret1 1.7. 3. J. Stat. Software.

[92] Bellégo C., Benatia D., Pape L. (2022). Dealing with logs and zeros in regression models. arXiv preprint arXiv:2203.11820.

[93] Aye M.S., Nomura H., Takahashi Y., Stringer L.C., Yabe M. (2022). Improving rice production efficiency in Myanmar by controlling for environmental production factors. J. Agric. Sci..

[94] Ducusin R.J.C., Espaldon M.V.O., De Guzman L.E.P., Rebancos C.M., Flores J.A. (2022). Adaptations toward climate risk: challenges and opportunities of the upland farmers in ifugao, Philippines. Int. J. Clim. Change Impacts Responses.

[95] Enovejas A.M., Maldia S., Komarudin N.A., Vergara D.G.K., Hilmi Y.S., Sevilla-Nastor J.B. (2021). Effect of climate variables in rice yield in nueva ecija, Philippines. Asia pacific journal of sustainable agriculture. Food and Energy.

[96] Chou J., Zhao W., Li J., Xu Y., Yang F., Sun M., Li Y. (2021). Changes in extreme climate events in rice-growing regions under different warming scenarios in China. Frontiers in earth science, 9.

[97] Osborn T.J., Jones P.D., Lister D.H., Morice C.P., Simpson I.R., Winn J.P., Hogan E., Harris I.C. (2021). Land surface air temperature variations across the globe updated to 2019: the CRUTEM5 data set. J. Geophys. Res. Atmos..

[98] Valipour M., Bateni S.M., Jun C. (2021). Global surface temperature: a new insight. Climate.

[99] Dai A. (2012). Increasing drought under global warming in observations and models. Nat. Clim. Change.

[100] Tsujimoto K., Kuriya N., Ohta T., Homma K., Im M. (2022). Quantifying the GCM-related uncertainty for climate change impact assessment of rainfed rice production in Cambodia by a combined hydrologic - rice growth model. Ecol. Model..

[101] Wu J., Han Z., Xu Y., Zhou B., Gao X. (2020). Changes in extreme climate events in China under 1.5 °C–4 °C global warming targets: projections using an ensemble of regional climate model simulations. J. Geophys. Res. Atmos..

[102] Hussain S., Huang J., Huang J., Ahmad S., Nanda S., Anwar S., Shakoor A., Zhu C., Zhu L., Cao X., Jin Q., Zhang J. (2020). Rice production under climate change: adaptations and mitigating strategies. Environment, climate. Plant and Vegetation Growth.

[103] Schneider P., Asch F. (2020). Rice production and food security in Asian Mega deltas—a review on characteristics, vulnerabilities and agricultural adaptation options to cope with climate change. J. Agron. Crop Sci..

[104] Gao L., Gao Q., Lorenc M. (2022). Comparison of total factor productivity of rice in China and Japan. Sustainability.

[105] Kamoshita A. (2007). Historical changes in urban rice production systems in tokyo, Japan. Plant Prod. Sci..

[106] Mayumi K.T. (2020). Reconsidering agriculture, forestry and fishery in Japan: searching for a responsible development pathway. Lect. Notes Eng..

[107] McGreevy S.R., Kobayashi M., Tanaka K. (2018). Agrarian pathways for the next generation of Japanese farmers. Can. J. Dev. Stud./Rev. Can. Études Dev..

[108] Usman M., Sawaya A., Igarashi M., Gayman J.J., Dixit R. (2021). Strained agricultural farming under the stress of youths' career selection tendencies: a case study from Hokkaido (Japan). Humanities and Soc. Sci. Commun..

[109] Khanal U., Wilson C., Hoang V.N., Lee B. (2018). Farmers' adaptation to climate change, its determinants and impacts on rice yield in Nepal. Ecol. Econ..

[110] Saediman H., La Ode Lasmin M.A.L., Rianse U., Geo L. (2020). Rice farmers' perception of climate variability in South konawe district of southeast sulawesi. Perception.

[111] van Oort P.A.J., Zwart S.J. (2017). Impacts of climate change on rice production in Africa and causes of simulated yield changes. Global Change Biol..

[112] Arnell N.W., Lowe J.A., Challinor A.J., Osborn T.J. (2019). Global and regional impacts of climate change at different levels of global temperature increase. Climatic Change.

[113] Dar M.H., Waza S.A., Shukla S., Zaidi N.W., Nayak S., Hossain M., Kumar A., Ismail A.M., Singh U.S. (2020). Drought tolerant rice for ensuring food security in eastern India. Sustainability.

[114] Pandey S., Byerlee D., Dawe D., Dobermann A., Mohanty S., Rozelle S., Hardy B. (2010). Rice in the Global Economy: Strategic Research and Policy Issues for Food Security.

[115] Doni F., Isahak A., Zain C.R.C.M., Yusoff W.M.W. (2014). Physiological and growth response of rice plants (Oryza sativa L.) to Trichoderma spp. inoculants. Amb. Express.

